# Alzheimer's disease drug development pipeline: 2019

**DOI:** 10.1016/j.trci.2019.05.008

**Published:** 2019-07-09

**Authors:** Jeffrey Cummings, Garam Lee, Aaron Ritter, Marwan Sabbagh, Kate Zhong

**Affiliations:** a Department of Brain Health, University of Nevada, Las Vegas (UNLV), School of Integrated Health Sciences, Las Vegas, NV, USA; b Cleveland Clinic Lou Ruvo Center for Brain Health, Las Vegas, NV, USA; c CNS Innovations, Henderson, NV, USA

**Keywords:** Alzheimer's disease, Drug development, Clinical trials, Biomarkers, Bayesian design, Adaptive design, Repurposed drugs

## Abstract

**Introduction:**

Alzheimer's disease (AD) has few available treatments, and there is a high rate of failure in AD drug development programs. Study of the AD drug development pipeline can provide insight into the evolution of drug development and how best to optimize development practices.

**Methods:**

We reviewed clinicaltrials.gov and identified all pharmacologic AD trials of all agents currently being developed for treatment of AD.

**Results:**

There are 132 agents in clinical trials for the treatment of AD. Twenty-eight agents are in 42 phase 3 trials; 74 agents are in 83 phase 2 trials; and 30 agents are in 31 phase 1 trials. There is an increase in the number of agents in each phase compared with that in the 2018 pipeline. Nineteen agents in trials target cognitive enhancement, and 14 are intended to treat neuropsychiatric and behavioral symptoms. There are 96 agents in disease modification trials; of these, 38 (40%) have amyloid as the primary target or as one of several effects. Eighteen of the antiamyloid agents are small molecules, and 20 are monoclonal antibodies or biological therapies. Seven small molecules and ten biologics have tau as a primary or combination target (18%). Amyloid is the most common specific target in phase 3 and phase 2 disease modification trials. Novel biomarkers (e.g., neurofilament light), new outcomes (e.g., AD Composite Score [ADCOMS]), enrollment of earlier populations, and innovative trial designs (e.g., Bayesian adaptive designs) are new features in recent clinical trials.

**Discussion:**

Drug development continues robustly at all phases despite setbacks in several programs in the recent past. Continuing unmet needs require a commitment to growing and accelerating the pipeline.

## 1 Introduction

Drug discovery and development for Alzheimer's disease (AD) is arduous. There have been no new drugs approved since 2003, and there are no approved disease-modifying treatments (DMTs) for AD. The challenges of drug development have become more complex as potential trial populations have expanded to include preclinical and prodromal AD, as well as AD dementia [1], [2], [3]. The US Food and Drug Administration (FDA) has provided guidance for clinical trials in AD dementia and predementia AD including use of a single primary outcome in trials of prodromal AD, the role of biomarkers in staging preclinical and prodromal AD, and the use of Bayesian statistics and adaptive clinical trial designs [4], [5], [6]. A new research framework for the diagnosis of AD based on amyloid, tau, and neurodegeneration (ATN) biomarkers was introduced by the National Institute on Aging (NIA) and the Alzheimer's Association [7]. This framework allows more precise classification of stages of AD, especially predementia stages, and may facilitate clinical trials of DMTs in AD [8]. Progress in biomarkers relevant to clinical trials of AD include increased understanding of the role of tau positron emission tomography (PET) in characterizing and staging AD and development of new fluid biomarkers such as neurofilament light and neurogranin that are increasingly integrated into clinical trials [9], [10]. These advances comprise the foundations for progress in drug development and demonstrate collaboration among key stakeholders including basic and translational neuroscientists, clinician-scientists, pharmacy benefit managers, regulators, the National Institutes of Health (NIH), advocacy groups, and participants and family members.

In our annual update on the state of the AD drug development pipeline, we build on prior contributions to discuss the current phase 1, phase 2, and phase 3 clinical trials in AD [11], [12], [13]. We describe clinical trials and experimental treatments for disease modification, cognitive enhancement, and neuropsychiatric symptoms of AD. We note changes from 2018 and discuss specific areas of interest including repurposed agents, immunotherapies, novel mechanisms, the use of biomarkers in drug development, and new trends in AD clinical trials. Our goal is to continuously learn from the drug development process, identify best practices, and provide an update and overview of the current state.

## 2 Methods

Clinicaltrials.gov provides the source of information for this review. There are other clinical trial registries, and our review does not represent an exhaustive listing of every clinical trial in AD. However, the “Common Rule” governing clinicaltrials.gov mandates registration of all trials from sponsors with an investigational new drug or investigational new device [14], [15] being assessed in the US. Compliance with the required trial registration is high [16], [17], [18]. The US has more clinical trials than any other nation, and thus clinicaltrials.gov includes most agents currently in clinical trials for AD.

**Fig. 1:**
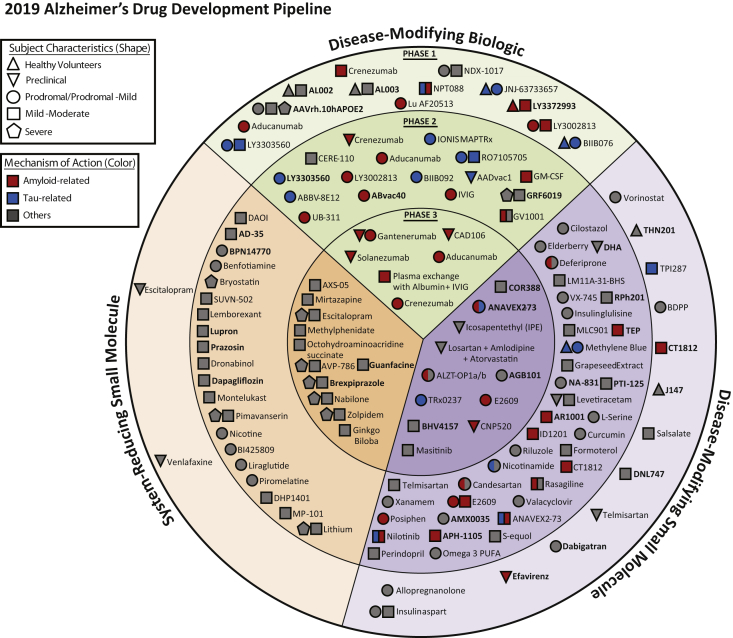
All compounds in AD clinical trials as of February 12, 2019 (the inner ring shows phase 3 agents; the middle ring is comprised of phase 2 agents; the outer ring presents phase 1 compounds; agents in green areas are biologics; agents in purple areas are disease-modifying small molecules; agents in orange areas are symptomatic agents addressing cognitive enhancement or behavioral and neuropsychiatric symptoms; the shape of the icon shows the population of the trial; the icon color shows the class of target for the agent.). Bolded names represent agents new to that phase since 2018.

We assayed clinicaltrials.gov as of February 12, 2019, and the tables and discussion provided apply to the information available at that time. We comment on terminated trials if the information has become publicly available but is not yet reflected on clinicaltrials.gov. We include all trials of all agents in phase 1, 2, and 3; if trials are presented as 1/2 or 2/3 in the clinicaltrials.gov database, we use that nomenclature in the review. Our trial database tracks trial title; trial number in clinicaltrials.gov; beginning date; projected end date; calculated trial duration; duration of treatment exposure; number of subjects planned for enrollment; number of arms of the study (usually a placebo arm and one or more treatment arms with different doses); whether a biomarker was described; subject characteristics; and sponsorship (a biopharmaceutical company, NIH, academic medical center, “other” entity such as a consortium or a philanthropic organization or a combination of these sponsors). We used the clinicaltrials.gov labeling and included trials that were recruiting, active but not recruiting (e.g., trials that have completed recruiting and are continuing with the exposure portion of the trial), enrolling by invitation, and not yet recruiting. We did not include trials listed as completed, terminated, suspended, unknown, or withdrawn. Information on these trials and reasons for their current status may not be publicly revealed. We do not include trials of nonpharmacologic therapeutic approaches such as cognitive therapies and caregiver interventions; we do not include studies of supplements and medical foods. We provide a table and brief discussion of new device trials (not included in Fig. 1). We do not include trials of biomarkers, although we note whether biomarkers were used in the trials reviewed. We include stem cell therapies among the interventions reviewed (not integrated into Fig. 1).

Drug targets and mechanisms of action (MOA) are important aspects of this review. MOA was determined from the information on clinicaltrials.gov or from a comprehensive search of the literature. In a few cases, the mechanism is undisclosed and could not be identified in the literature; we note these agents as having an “unknown” or “undisclosed” MOA. We grouped the mechanisms into symptomatic agents or DMTs. We divided the symptomatic agents into those that are putative cognitive enhancing agents or those that address neuropsychiatric and behavioral symptoms. DMTs were divided into small molecules or biologics including immunotherapies. DMTs were further divided into those targeting amyloid-related mechanisms, those that have tau-related MOAs, and those with “other” mechanisms such as neuroprotection, anti-inflammatory effects, growth factor promotion, or metabolic effects. The distinction between symptomatic and disease-modifying agents can be arbitrary, and some agents may have both properties. For purposes of this review, we chose what appears to be the principal MOA.

## 3 Results

### 3.1 Overview

As of February 12, 2019, there were 132 agents in 156 trials of anti-AD therapies. Fig. 1 shows the universe of pharmacologic compounds currently in clinical trials for AD. Nineteen (14%) agents in trials target cognitive enhancement, and 14 (11%) are intended to treat neuropsychiatric and behavioral symptoms. There are 96 (73%) agents that intend to achieve disease modification; 38 (40%) of these have amyloid; and 17 (18%) have tau as the primary target or as one of several effects seen in nonclinical studies. Eighteen of the antiamyloid agents are small molecules, and 20 are monoclonal antibodies or biological therapies. Anti-tau agents include seven small molecules and ten biologics.

All compounds in AD clinical trials as of February 12, 2019 (the inner ring shows phase 3 agents; the middle ring is comprised of phase 2 agents; the outer ring presents phase 1 compounds; agents in green areas are biologics; agents in purple areas are disease-modifying small molecules; agents in orange areas are symptomatic agents addressing cognitive enhancement or behavioral and neuropsychiatric symptoms; the shape of the icon shows the population of the trial; the icon color shows the class of target for the agent.). Bolded names represent agents new to that phase since 2018.

### 3.2 Phase 3

**Fig. 2:**
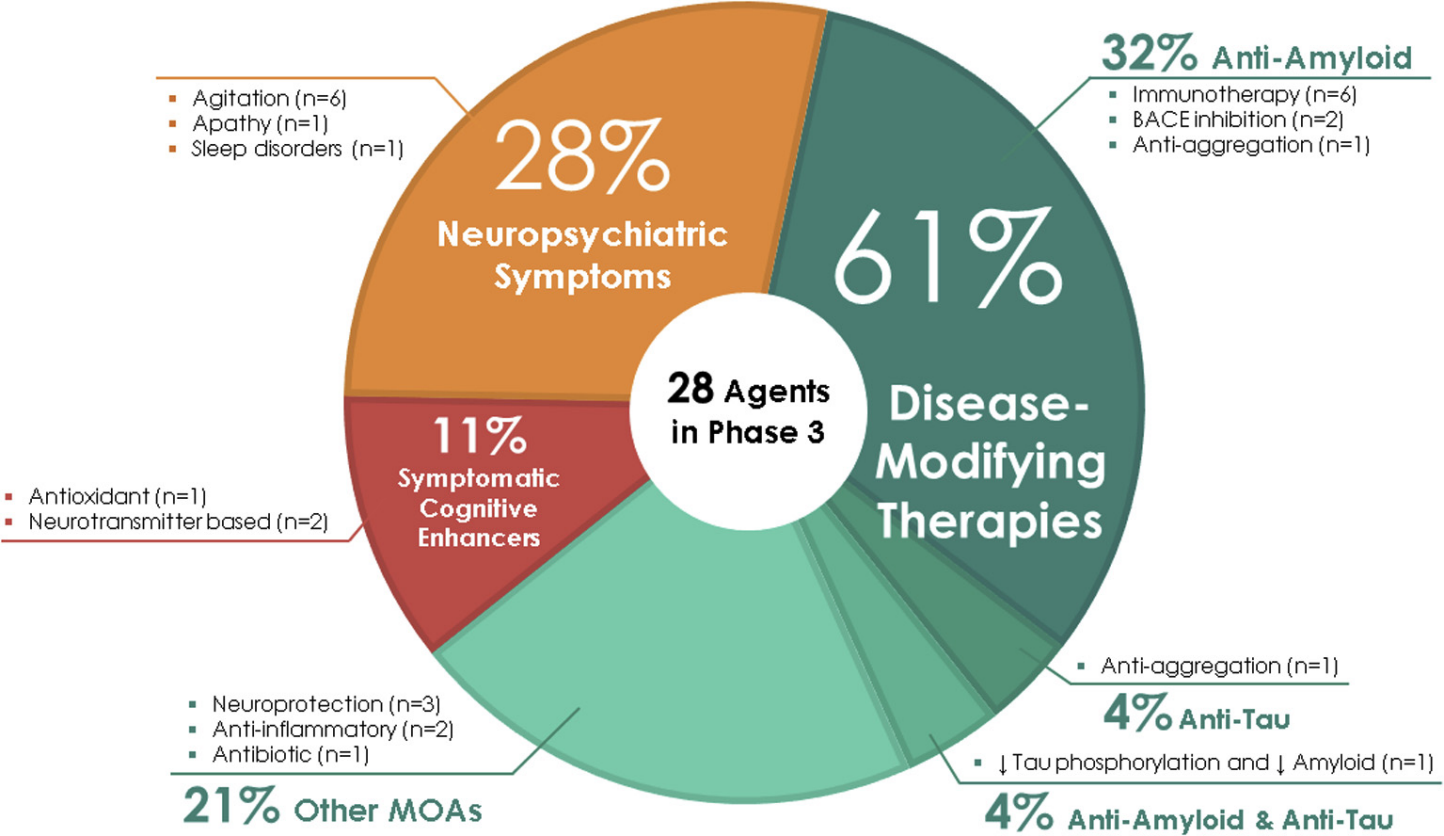
Mechanisms of action of agents in phase 3.

In phase 3, there are 28 agents in 42 trials (Figs. 1 and 2, Table 1). There are 11 symptomatic agents in phase 3; three cognitive enhancers and eight targeting behavioral symptoms. There are six biological therapies and 11 oral agents/small molecules in phase 3 that target disease modification. All the biological therapies and four of the small molecules have amyloid as the primary or one of several targets. There is one anti-tau agent in phase 3: LMTX (TRx0237). A phase 3 trial of this agent failed to show a drug-placebo difference [19], and based on the results, a new phase 2/3 trial (LUCIDITY) was started in 2018 with a lower dose of LMTX as monotherapy. Other mechanisms represented among phase 3 DMT molecules include neuroprotection, anti-inflammatory approaches, and metabolic interventions. Of the DMTs, two are repurposed agents approved for use in another indication (losartan plus amlodipine plus atorvastatin; and levetiracetam). Of the drugs with amyloid targets, there were six biologics, two beta-site amyloid precursor protein cleavage enzyme (BACE) inhibitors, and one anti-aggregation agent. Fig. 2 shows the MOAs of agents in phase 3.

**Table 1:** Agents currently in phase 3 of Alzheimer's disease drug development (as of February 12, 2019)

Agent	Agent mechanism class	Mechanism of action	Therapeutic purpose	ClinicalTrials.gov ID	Status	Sponsor	Start date	Estimated end date
Aducanumab∗	Antiamyloid	Monoclonal antibody directed at plaque and oligomers	Remove amyloid (DMT)	NCT02484547	Active, not recruiting	Biogen	Sep 2015	Apr 2022
				NCT02477800	Active, not recruiting	Biogen	Aug 2015	Apr 2022
**AGB101** (low-dose levetiracetam)	Neuroprotective	SV2A modulator	Decrease amyloid-induced neuronal hyperactivity (DMT)	NCT03486938	Recruiting	AgeneBio, NIA	Jan 2019	Nov 2022
Plasma exchange with albumin + immunoglobulin∗	Antiamyloid	Plasma exchange	Remove amyloid (DMT)	NCT01561053†	Active, not recruiting	Grifols	Mar 2012	Dec 2017
ALZT-OP1a + ALZT-OP1b (cromolyn + ibuprofen)	Antiamyloid, anti-inflammatory	Mast cell stabilizer (cromolyn), anti-inflammatory (ibuprofen)	Reduce neuronal damage; mast cells may also play a role in amyloid pathology (DMT)	NCT02547818	Recruiting	AZTherapies, Pharma Consulting Group, KCAS Bio, APCER Life Sciences	Sep 2015	Nov 2019
**ANAVEX2-73**	Anti-tau, antiamyloid, anti-inflammatory	Sigma-1 receptor agonist (high affinity), muscarinic agonist (low affinity), GSK-3β inhibitor	Improve cell signaling (cognitive enhancer) and reduce tau phosphorylation and amyloid (DMT)	NCT03790709†	Recruiting	Anavex Life Sciences	Jul 2018	Mar 2021
AVP-786	Neurotransmitter based	Sigma-1 receptor agonist; NMDA receptor antagonist	Improve neuropsychiatric symptoms (agitation)	NCT02442765	Active, not recruiting	Avanir	Sep 2015	Apr 2019
				NCT02442778	Recruiting	Avanir	Sep 2015	Dec 2019
				NCT02446132	Recruiting, extension	Avanir	Dec 2015	Jun 2022
				NCT03393520	Recruiting	Avanir	Oct 2017	Jun 2021
AXS-05	Neurotransmitter based	Sigma-1 receptor agonist; NMDA receptor antagonist (dextromethorphan); dopamine-norepinephrine reuptake inhibitor (bupropion)	Improve neuropsychiatric symptoms (agitation)	NCT03226522†	Recruiting	Axsome Therapeutics	Jul 2017	Sep 2019
**BHV4157** (troriluzole)	Neuroprotective	Glutamate modulator	Reduce synaptic levels of glutamate (DMT)	NCT03605667†	Recruiting	Biohaven Pharma, ADCS	Jul 2018	Feb 2020
**Brexpiprazole**	Neurotransmitter based	Atypical antipsychotic; D2 receptor partial agonist and serotonin-dopamine modulator	Improve neuropsychiatric symptoms (agitation)	NCT03620981†	Recruiting	Otsuka	Aug 2018	Nov 2021
				NCT03594123	Recruiting, extension	Otsuka	Oct 2018	Aug 2021
				NCT03548584	Recruiting	Otsuka	May 2018	Dec 2020
				NCT03724942	Recruiting, extension	Otsuka	Nov 2018	May 2021
CAD106 & CNP520	Antiamyloid	Amyloid vaccine (CAD106), BACE inhibitor (CNP520)	Remove amyloid (vaccine); prevent amyloid production (BACE inhibitor) (DMT)	NCT02565511†	Recruiting	Novartis, Amgen, NIA, Alzheimer's Association, Banner Alzheimer's Institute	Feb 2016	Jan 2025
CNP520	Antiamyloid	BACE inhibitor	Prevent amyloid production (DMT)	NCT03131453†	Recruiting	Novartis, Amgen, Banner Alzheimer's Institute	Aug 2017	Mar 2025
**COR388**	Anti-inflammatory	Bacterial protease inhibitor targeting a periodontal pathogen	Reduce neuroinflammation and hippocampal degeneration (DMT)	NCT03823404†	Not yet recruiting	Cortexyme	Apr 2019	Dec 2022
Crenezumab∗	Antiamyloid	Monoclonal antibody directed at oligomers	Remove amyloid (DMT)	NCT02670083	Active, not recruiting	Roche	Mar 2016	Jul 2021
				NCT03114657	Recruiting	Roche	Mar 2017	Oct 2022
				NCT03491150	Recruiting, extension	Roche	Apr 2018	Nov 2022
E2609 (elenbecestat)	Antiamyloid	BACE inhibitor	Reduce amyloid production (DMT)	NCT02956486	Recruiting	Eisai, Biogen	Oct 2016	Jun 2021
				NCT03036280	Recruiting	Eisai, Biogen	Dec 2016	Jun 2021
Escitalopram	Neurotransmitter based	Serotonin reuptake inhibition	Improve neuropsychiatric symptoms (agitation)	NCT03108846	Recruiting	NIA, JHSPH Center for Clinical Trials	Jan 2018	Aug 2022
Gantenerumab	Antiamyloid	Monoclonal antibody	Remove amyloid (DMT)	NCT02051608	Active, not recruiting	Roche	Mar 2014	Nov 2020
				NCT01224106	Active, not recruiting	Roche	Nov 2010	Aug 2020
				NCT03444870	Recruiting	Roche	Jun 2018	May 2023
				NCT03443973	Recruiting	Roche	Jun 2018	May 2023
Gantenerumab & Solanezumab	Antiamyloid	Monoclonal antibody directed at plaque and oligomers (gantenerumab); Monoclonal antibody directed at monomers (solanezumab)	Remove amyloid/reduce amyloid production (DMT)	NCT01760005†	Active, not recruiting	Washington University, Eli Lilly, Roche, NIA, Alzheimer's Association	Dec 2012	Dec 2023
Ginkgo Biloba	Metabolic	Plant extract with antioxidant properties	Improve brain blood flow and mitochondrial function (cognitive enhancer)	NCT03090516†	Recruiting	Nanjing Medical University	Aug 2016	Mar 2018
**Guanfacine**	Neurotransmitter based	Alpha-2 adrenergic agonist	Modulation of noradrenergic deficit (cognitive enhancer)	NCT03116126	Not yet recruiting	Imperial College London, UK National Institute of Health Research	Sep 2018	Sep 2019
Icosapent ethyl (IPE)	Neuroprotective	Purified form of the omega-3 fatty acid EPA	Protect neurons from disease pathology (DMT)	NCT02719327†	Recruiting	VA Office of Research and Development, University of Wisconsin, Madison	Jun 2017	Nov 2021
Losartan & Amlodipine & Atorvastatin + exercise	Anti-inflammatory, metabolic	Angiotensin II receptor blocker (losartan), calcium channel blocker (amlodipine), cholesterol agent (atorvastatin)	Intensive vascular risk reduction can preserve cognitive function (DMT)	NCT02913664†	Recruiting	University of Texas Southwestern	Sep 2016	Sep 2022
Masitinib	Anti-inflammatory	Selective tyrosine kinase inhibitor	Activity on mast cells, modulation of inflammatory processes (DMT)	NCT01872598	Active, not recruiting	AB Science	Jan 2012	Oct 2019
Methylphenidate	Neurotransmitter based	Dopamine reuptake inhibitor	Improve neuropsychiatric symptoms (apathy)	NCT02346201	Recruiting	Johns Hopkins, NIA	Jan 2016	Aug 2020
Mirtazapine	Neurotransmitter based	Alpha-1 antagonist	Improve neuropsychiatric symptoms (agitation)	NCT03031184	Recruiting	University of Sussex	Jan 2017	Jul 2020
Nabilone∗	Neurotransmitter based	Cannabinoid (receptor agent)	Improve neuropsychiatric symptoms (agitation)	NCT02351882†	Active, not recruiting	Sunnybrook Health Sciences Center	Jan 2015	Mar 2019
Octohydroaminoacridine Succinate	Neurotransmitter based	Acetylcholinesterase inhibitor	Improve acetylcholine signaling (cognitive enhancer)	NCT03283059	Recruiting	Shanghai Mental Health Center, Changchun-Huayang High-tech Co., Jiangsu Sheneryang High-tech Co.	Aug 2017	Feb 2020
Solanezumab	Antiamyloid	Monoclonal antibody directed at monomers	Remove amyloid and prevent aggregation (DMT)	NCT02008357	Active, not recruiting	Eli Lilly, ATRI	Feb 2014	Jul 2022
TRx0237 (LMTX)	Anti-tau	Tau protein aggregation inhibitor	Reduce tau-mediated neuronal damage (DMT)	NCT03446001†	Recruiting	TauRx Therapeutics	Jan 2018	Jun 2020
Zolpidem	Neurotransmitter based	Positive allosteric modulator of GABA-A receptors	Improve neuropsychiatric symptoms (sleep disorders)	NCT03075241	Recruiting	Brasilia University Hospital	Oct 2016	Dec 2018

Abbreviations: ATRI, Alzheimer's Therapeutic Research Institute; BACE, beta-site amyloid precursor protein cleaving enzyme; DMT, disease-modifying therapy; EPA, eicosapentaenoic acid; GABA, gamma-aminobutyric acid; GSK, glycogen synthase kinase; NIA, National Institute on Aging; SV2A, synaptic vesicle protein 2A.

NOTE. Twenty-eight agents in 42 phase 3 clinical trials currently ongoing as of February 12, 2019 according to clinicaltrials.gov.

Bolded terms represent new agents into the 2019 phase 3 pipeline.

∗ Reported as terminated or completed after the data collection date of February 12, 2019.

† Phase 2/3 trials.

There were six prevention trials enrolling cognitively normal participants; 14 trials in patients with prodromal AD/mild cognitive impairment (MCI) or prodromal-to-mild AD; 12 trials of patients with mild-to-moderate AD; and 10 trials of patients with mild-to-severe AD.

Phase 3 trials included an average of 640 participants and had a mean duration of 246 weeks (including the recruitment and the treatment period). Mean treatment exposure period was 73 weeks. DMT trials were longer and larger than trials of symptomatic agents with a mean duration of 297 weeks including 112 treatment weeks, and included an average of 862 participants. The mean duration of cognitive enhancer trials was 88 weeks (17 treatment weeks), and they included an average of 333 participants. Trials of agents for behavioral symptoms had a mean duration of 187 weeks (15 treatment weeks) and included a mean of 311 subjects.

The average duration of treatment exposure for phase 3 DMTs is 112 weeks, and the mean period from trial initiation to primary completion date (final data collection date for primary outcome measures) is 269 weeks. This indicates that 157 weeks, more than the treatment period, is the average anticipated recruitment time. When examined by trial population, DMT prevention trials are 405 weeks in duration (192 treatment weeks); trials for patients with MCI/prodromal/prodromal-to-mild AD are 263 weeks in duration (98 treatment weeks); and trials for patients with mild-to-moderate AD are 264 weeks in duration (57 treatment weeks). Planned recruitment periods for these three types of trials are 192, 130, and 191 weeks, respectively.

### 3.3 Phase 2

**Fig. 3:**
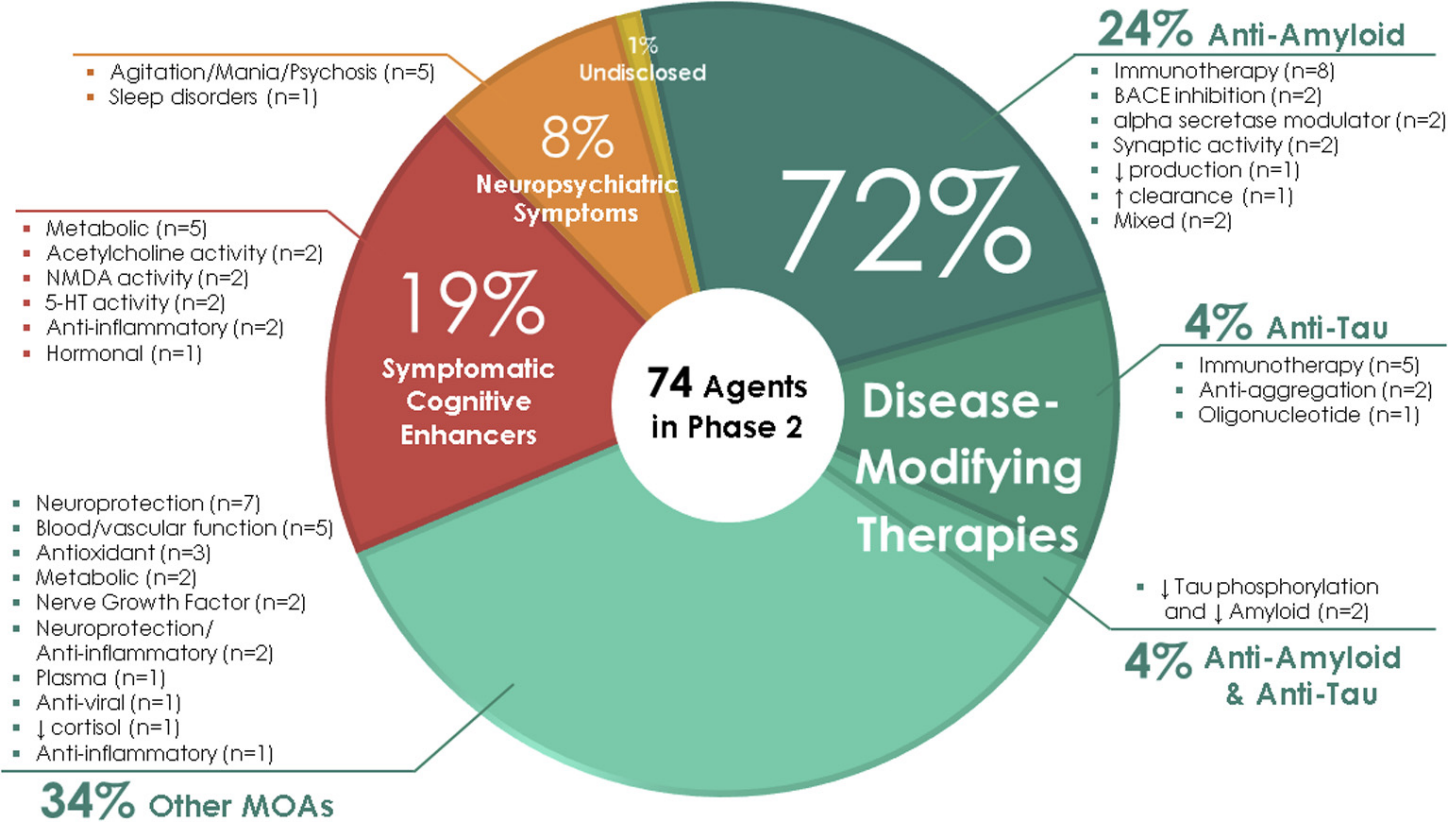
Mechanisms of action of agents in phase 2.

Phase 2 has a larger array of therapies and mechanisms that are being assessed than are represented in phase 3. There are 74 agents in 83 trials (Figs. 1 and 3, Table 2). Of these, there are 20 symptomatic agents; 14 cognitive enhancers; and six agents targeting behavioral symptoms. There are 53 potential disease-modifying agents in phase 2 trials; 16 biologics and 37 small molecules. One agent had an undisclosed mechanism. Twelve of the small molecules and eight of the biologics have amyloid reduction as one of the mechanisms observed in nonclinical studies (38% of DMTs). Four small molecules and six biologics in phase 2 target tau as one of their mechanisms (19% of DMTs). There are 24 small molecules and two biologics with neuroprotection as one of the mechanisms (49% of DMTs). Other mechanisms represented in phase 2 include anti-inflammatory and metabolic interventions as the primary or one of a combination of effects documented in animal models. There are six trials involving stem cell therapies. Sixteen of the DMT agents are repurposed agents approved for use in another indication.

**Table 2:** Agents currently in phase 2 of Alzheimer's disease drug development (as of February 12, 2019)

Agent	Agent mechanism class	Mechanism of action	Therapeutic purpose	ClinicalTrials.gov ID	Status	Sponsor	Start date	Estimated end date
AADvac1	Anti-tau	Active immunotherapy	Remove tau and prevent tau propagation (DMT)	NCT02579252	Active, not recruiting	Axon Neuroscience	Mar 2016	Jun 2019
ABBV-8E12	Anti-tau	Monoclonal antibody	Remove tau and prevent tau propagation (DMT)	NCT02880956	Recruiting	AbbVie	Oct 2016	Sep 2022
				NCT03712787	Not yet recruiting, extension	AbbVie	Nov 2018	Aug 2027
**ABvac40**	Antiamyloid	Active immunotherapy	Remove amyloid (DMT)	NCT03461276	Recruiting	Araclon Biotech	Feb 2018	Feb 2021
**AD-35**	Neurotransmitter based	Acetylcholinesterase inhibitor	Improve acetylcholine signaling (cognitive enhancer)	NCT03625401	Recruiting	Zhejiang Hisun Pharmaceutical, Medpace, Inc.	Oct 2018	Jul 2020
				NCT03790982	Active, not recruiting	Zhejiang Hisun Pharmaceutical	Dec 2018	Jul 2021
Aducanumab∗	Antiamyloid	Monoclonal antibody directed at plaque and oligomers	Remove amyloid (DMT)	NCT03639987	Recruiting	Biogen	Dec 2018	Nov 2023
**AMX0035**	Neuroprotective	Blocks mitochondrial and endoplasmic reticulum stress	Blocks nerve cell death and neuroinflammation (DMT)	NCT03533257	Recruiting	Amylyx Pharmaceuticals, ADDF, Alzheimer's Association	Aug 2018	Sep 2020
ANAVEX 2-73	Anti-tau, antiamyloid, anti-inflammatory	Sigma-1 receptor agonist (high affinity); muscarinic agonist (low affinity); GSK-3β inhibitor	Improve cell signaling (cognitive enhancer) and reduce tau phosphorylation and amyloid (DMT)	NCT02756858	Active, not recruiting, extension	Anavex Life Sciences	Mar 2016	Nov 2020
**APH-1105**	Antiamyloid	Alpha-secretase modulator	Reduce amyloid (DMT)	NCT03806478	Not yet recruiting	Aphios	Jun 2021	Dec 2022
**AR1001**	Antiamyloid	PDE 5 inhibitor	Improve synaptic plasticity and reduce amyloid (DMT)	NCT03625622	Recruiting	AriBio Co.	Jan 2019	Aug 2020
AstroStem	Regenerative	Stem cell therapy; autologous adipose tissue derived mesenchymal stem cells	Regenerate neurons (DMT)	NCT03117738†	Recruiting	Nature Cell Co.	Apr 2017	Jul 2019
BAC	Undisclosed	Undisclosed	Undisclosed	NCT02886494	Recruiting	Charsire Biotechnology	Dec 2016	Nov 2019
				NCT02467413	Not yet recruiting	Charsire Biotechnology, A2 Healthcare Taiwan Corporation	Dec 2019	Dec 2021
Benfotiamine	Metabolic	Synthetic thiamine (B1)	Improve multiple cellular processes (cognitive enhancer)	NCT02292238	Recruiting	Burke Medical Research Institute, Columbia University, NIA, ADDF	Nov 2014	Nov 2019
BI425809	Neurotransmitter based	Glycine transporter 1 inhibitor	Facilitate NMDA receptor activity (cognitive enhancer)	NCT02788513	Recruiting	Boehringer Ingelheim	Aug 2016	Mar 2020
BIIB092	Anti-tau	Monoclonal antibody	Remove tau and reduce tau propagation (DMT)	NCT03352557	Recruiting	Biogen	May 2018	Jul 2021
**BPN14770**	Anti-inflammatory	PDE4D inhibitor	Prolongs cAMP activity (cognitive enhancer)	NCT03817684	Not yet recruiting	Tetra Discovery Partners	Apr 2019	Jun 2020
Byrostatin	Metabolic	Protein kinase C modulator	Improve multiple cellular processes (cognitive enhancer)	NCT03560245	Recruiting	Neurotrope Bioscience	Jun 2018	Jul 2019
Candesartan	Neuroprotective, metabolic, antiamyloid	Angiotensin receptor blocker	Improve vascular functioning and reduce amyloid (DMT)	NCT02646982	Recruiting	Emory University	Jun 2016	Sep 2021
CERE-110∗	Neuroprotective	Adeno-associated virus-based gene delivery vector of nerve growth factor	Cholinergic neuronal hypertrophy; slows age-related neurodegeneration (DMT)	NCT00876863	Active, not recruiting	Sangamo Therapeutics, ADCS	Sep 2009	Mar 2020
Cilostazol	Neuroprotective	PDE-3 inhibitor	Reduce accumulation of amyloid and reduce tau phosphorylation; improve cerebral circulation (DMT)	NCT02491268	Recruiting	National Cerebral and Cardiovascular Center, Japan	Jul 2015	Dec 2020
Crenezumab∗	Antiamyloid	Monoclonal antibody targeting soluble oligomers	Remove amyloid (DMT)	NCT01998841	Active, not recruiting	Genentech, NIA Banner Alzheimer's Institute	Dec 2013	Feb 2022
CT1812	Antiamyloid	Sigma-2 receptor antagonist	Reduce amyloid-beta protein-induced synaptic toxicity (DMT)	NCT03507790	Recruiting	Cognition Therapeutics	Oct 2018	Dec 2019
				NCT03493282†	Recruiting	Cognition Therapeutics	Apr 2018	Jan 2020
Curcumin + aerobic yoga	Neuroprotective	Herb with antioxidant and anti-inflammatory properties	Reduce amyloid production, decrease neuroglial cell proliferation (DMT)	NCT01811381	Recruiting	VA Office of Research and Development	Jan 2014	Dec 2019
DAOI	Neurotransmitter based	NMDA receptor modulation	Enhance NMDA activity (cognitive enhancer)	NCT03752463	Recruiting	Chang Gung Memorial Hospital, Taiwan	May 2015	Dec 2019
**Dapagliflozin**	Metabolic	SGLT2 inhibitor	Improve insulin sensitivity (cognitive enhancer)	NCT03801642†	Recruiting	University of Kansas	Feb 2019	Oct 2020
Deferiprone	Antiamyloid, neuroprotective	Iron chelating agent	Reduce reactive oxygen species that damage neurons; effect on amyloid and BACE pathology (DMT)	NCT03234686	Recruiting	Neuroscience Trials Australia	Jan 2018	Dec 2021
**DHA**	Neuroprotective	Omega-3 fatty acid in high concentration in the brain	Reduce amyloid production, improve synaptic function (DMT)	NCT03613844	Recruiting	University of Southern California	Jul 2018	Sep 2024
DHP1401	Metabolic	Affects cAMP activity	Improve synaptic function (cognitive enhancer)	NCT03055741	Active, not recruiting	Daehwa Pharmaceutical Co.	Dec 2016	Jun 2019
Dronabinol	Neurotransmitter based	CB1 and CB2 endocannabinoid receptor partial agonist	Improve neuropsychiatric symptoms (agitation)	NCT02792257	Recruiting	Mclean Hospital, Johns Hopkins University	Mar 2017	Dec 2020
E2609 (elenbecestat)	Antiamyloid	BACE inhibitor	Reduce amyloid production (DMT)	NCT02322021	Active, not recruiting	Eisai, Biogen	Nov 2014	Jun 2020
Elderberry Juice	Anti-inflammatory, neuroprotective	Antioxidant rich in anthocyanins	Improve mitochondrial function (DMT)	NCT02414607†	Recruiting	University of Missouri	Sep 2016	Apr 2019
Formoterol	Metabolic	Beta-2 adrenergic receptor agonist	Effects on multiple cellular pathways (DMT)	NCT02500784	Recruiting	Palo Alto Veterans Institute for Research, Mylan, Alzheimer's Association	Jan 2015	Jul 2018
Grapeseed Extract	Neuroprotective	Polyphenolic compounds; antioxidant	Anti-oligomerization agent; prevents aggregation of amyloid and tau (DMT)	NCT02033941	Recruiting	Mount Sinai School of Medicine, NCCIH	Nov 2014	Sep 2018
**GRF6019**	Anti-inflammatory	Human plasma protein fraction infusions	Young blood parabiosis can counteract inflammatory and age-related processes in the brain (DMT)	NCT03520998	Recruiting	Alkahest	Apr 2018	Nov 2019
				NCT03765762	Recruiting	Alkahest	Dec 2018	Nov 2019
GV1001	Antiamyloid, metabolic	Telomerase reverse transcriptase peptide vaccine	Effects on multiple cellular pathways including amyloid pathology (DMT)	NCT03184467	Recruiting	GemVax & Kael	Jun 2017	Jun 2019
hUCB-MSCs	Regenerative	Stem cell therapy	Regenerate neurons; reduce amyloid plaque deposition and soluble amyloid; decrease microglial systemic inflammation (DMT)	NCT02054208†	Recruiting	Medipost Co.	Feb 2014	Jul 2019
				NCT03172117†	Recruiting, extension	Medipost Co.	May 2017	Dec 2021
				NCT02513706	Ongoing	South China Research Center	Oct 2017	Oct 2019
				NCT02672306†	Ongoing	South China Research Center	Oct 2017	Oct 2019
				NCT02833792	Active, not recruiting	Stemedica Cell Technologies	Jun 2016	Jun 2020
ID1201	Antiamyloid	Alpha-secretase enhancer	Reduce amyloid (DMT)	NCT03363269	Ongoing	IlDong Pharmaceutical	Apr 2016	Dec 2018
Insulin glulisine (intranasal)	Metabolic	Increase insulin signaling in the brain	Enhance cell signaling and growth; promote neuronal metabolism (DMT)	NCT02503501	Ongoing	HealthPartners Institute	Aug 2015	May 2019
IONIS MAPTRx (BIIB080)	RNA-based anti-tau	MAPT RNA inhibitor; antisense oligonucleotide	Reduce tau production (DMT)	NCT03186989†	Recruiting	Ionis Pharmaceuticals, Biogen	Jun 2017	Feb 2020
Lemborexant	Neurotransmitter based	Dual antagonist of orexin OX1 and OX2 receptors	Improve neuropsychiatric symptoms (sleep disorders)	NCT03001557	Active, not recruiting	Eisai, Purdue	Dec 2016	Apr 2020
Levetiracetam	Neuroprotective	SV2A modulator	Decrease amyloid-induced neuronal hyperactivity (DMT)	NCT02002819	Recruiting	University of California, San Francisco	Jun 2014	Dec 2019
				NCT03489044	Recruiting	University of Oxford, NHS Foundation Trust, UCB Pharma	Nov 2018	Jan 2020
				NCT03461861	Recruiting	Medical College of Wisconsin, NIA	Nov 2018	Mar 2019
Liraglutide	Metabolic	Glucagon-like peptide 1 receptor agonist	Enhance cell signaling (cognitive enhancer)	NCT01843075	Recruiting	Imperial College London	Jan 2014	Mar 2019
Lithium	Neurotransmitter based	Ion channel modulator	Improve neuropsychiatric symptoms (agitation, mania, psychosis)	NCT02129348	Recruiting	New York State Psychiatric Institute, NIA	Jun 2014	Apr 2019
LM11A-31-BHS	Neuroprotective	p75 neurotrophin receptor ligand	Inhibits tau phosphorylation and synaptic dysfunction; prevents amyloid-induced toxicity (DMT)	NCT03069014†	Recruiting	PharmatrophiX Inc., NIA	Feb 2017	Oct 2019
**Lupron** (leuprolide acetate depot)	Metabolic	Gonadotropin-releasing hormone receptor agonist	Suppresses brain-produced gonadotropin-releasing hormone (cognitive enhancer)	NCT03649724	Not yet recruiting	New York University	Dec 2018	Dec 2020
L-Serine	Neuroprotective	Amino acid	Stabilizes protein misfolding (DMT)	NCT03062449	Recruiting	Dartmouth-Hitchcock Medical Center, Brain Chemistry Laboratories	Mar 2017	Aug 2019
LY3002813	Antiamyloid	Monoclonal antibody	Remove amyloid (DMT)	NCT03367403	Recruiting	Eli Lilly	Dec 2017	Sep 2021
**LY3303560**	Anti-tau	Monoclonal antibody	Remove tau and reduce tau propagation (DMT)	NCT03518073	Recruiting	Eli Lilly	Apr 2018	Oct 2021
Methylene blue	Anti-tau	Tau protein aggregation inhibitor	Reduce neurofibrillary tangle formation (DMT)	NCT02380573	Active, not recruiting	Texas Alzheimer's Research and Care Consortium	Jul 2015	Jul 2019
MLC901 (NeuroAiD)	Neuroprotective, anti-inflammatory	Traditional Chinese medicine consisting of several herbs	Multiple cellular pathways (DMT)	NCT03038035	Recruiting	National University Hospital, Singapore	Dec 2016	Jun 2019
Montelukast	Anti-inflammatory	Leukotriene receptor antagonist	Reduce inflammatory pathways (cognitive enhancer)	NCT03402503	Recruiting	IntelGenx Corp.	Nov 2018	Oct 2020
MP-101	Neurotransmitter based	Enhance mitochondrial functioning	Improve neuropsychiatric symptoms (psychosis)	NCT03044249	Recruiting	Mediti Pharma	May 2017	Jan 2021
**NA-831** (traneurocin)	Neuroprotective	Undisclosed	Neurogenesis and neuroprotection (DMT)	NCT03538522	Not yet recruiting	NeuroActiva	Sep 2018	Apr 2019
Neflamapimod (VX-745)	Anti-inflammatory	Selective p38 MAPK inhibitor	Affects multiple cellular processes including inflammation and cellular plasticity; reduces amyloid plaque burden (DMT)	NCT03402659	Recruiting	EIP Pharma, VU University	Dec 2017	Jul 2019
				NCT03435861	Recruiting	EIP Pharma, Toulouse University, Foundation Plan Alzheimer	Oct 2018	Jan 2021
Nicotinamide	Anti-tau, neuroprotective	Histone deacetylase inhibitor	Reduce tau-induced microtubule depolymerization (DMT)	NCT03061474	Recruiting	University of California, Irvine	Jul 2017	Feb 2019
Nicotine	Neurotransmitter based	Nicotinic acetylcholine receptor agonist	Enhance acetylcholine signaling (cognitive enhancer)	NCT02720445	Recruiting	Univ. of Southern California, NIA, ATRI, Vanderbilt University	Jan 2017	Dec 2019
Nilotinib	Antiamyloid, anti-tau	Tyrosine kinase inhibitor	Reduce amyloid and tau phosphorylation (DMT)	NCT02947893	Active, not recruiting	Georgetown University	Jan 2017	Dec 2019
Octagam 10%	Antiamyloid	10% human normal immunoglobulin	Remove amyloid (DMT)	NCT03319810	Recruiting	Sutter Health	Jan 2018	May 2019
Omega-3 PUFA	Neuroprotective	Fish oil concentrate standardized to long chain in n-3 PUFA content	Support small blood vessels in the brain (DMT)	NCT01953705	Active, not recruiting	Oregon Health and Science University, NIA	May 2014	Sep 2019
Pimavanserin	Neurotransmitter based	5-HT2A inverse agonist	Improve neuropsychiatric symptoms (psychosis)	NCT03118947	Active, not recruiting, extension	Acadia	Feb 2017	Aug 2019
Piromelatine	Neurotransmitter based	Melatonin receptor agonist; 5-HT 1A and 1D serotonin receptor agonist	Enhance cellular signaling (cognitive enhancer)	NCT02615002	Recruiting	Neurim Pharmaceuticals	Nov 2015	Apr 2019
Posiphen	Antiamyloid	Selective inhibitor of APP production	Reduce amyloid production (DMT)	NCT02925650†	Recruiting	QR Pharma, ADCS	Mar 2017	Dec 2019
**Prazosin**	Neurotransmitter based	Alpha-1 adrenoreceptor antagonist	Improve neuropsychiatric symptoms (agitation)	NCT03710642	Recruiting	ADCS, NIA	Jan 2019	Dec 2022
**PTI-125**	Neuroprotective, anti-inflammatory	FLNA inhibitor	Reduce amyloid, prevent tau hyperphosphorylation and inflammatory toxicity (DMT)	NCT03748706	Recruiting	Pain Therapeutics, NIH	Nov 2018	Mar 2019
Rasagiline∗	Antiamyloid, neuroprotective, metabolic	Monoamine oxidase B inhibitor	Enhance mitochondria activity and inactivate reactive oxygen species (cognitive enhancer), also effect on amyloid pathology (DMT)	NCT02359552	Active, not recruiting	The Cleveland Clinic	May 2015	Feb 2019
Riluzole	Neuroprotective	Glutamate receptor antagonist	Inhibit glutamate neurotransmission (DMT)	NCT01703117	Recruiting	Rockefeller University	Nov 2013	Nov 2019
RO7105705 (MTAU9937 A)	Anti-tau	Monoclonal antibody	Remove tau (DMT)	NCT03289143	Recruiting	Genentech	Oct 2017	Sep 2022
				NCT03828747	Recruiting	Genentech	Feb 2019	Sep 2021
**RPh201**	Neuroprotective	Undisclosed	Promote neurogenesis (DMT)	NCT03462121	Recruiting	Regenera Pharma	Mar 2018	Apr 2019
Sargramostim∗ (GM-CSF)	Antiamyloid, neuroprotective	Synthetic granulocyte colony stimulator	Stimulate innate immune system to remove amyloid pathology; increase neuronal connectivity (DMT)	NCT01409915	Active, not recruiting	University of Colorado, Denver, The Dana Foundation	Mar 2011	Nov 2019
S-equol (AUS-131)	Neuroprotective	Nonhormonal estrogen receptor B agonist	Mitochondrial function potentiation; improve synaptic functioning, protects neurons (DMT)	NCT03101085†	Recruiting	Ausio Pharmaceuticals, University of Kansas	May 2017	Oct 2019
SUVN-502	Neurotransmitter based	5-HT 6 antagonist	Improve neuronal signaling (cognitive enhancer)	NCT02580305	Active, not recruiting	Suven Life Sciences	Sep 2015	May 2019
Telmisartan & Perindopril	Neuroprotective, anti-inflammatory	Angiotensin II receptor blocker, PPAR-gamma agonist (telmisartan); angiotensin converting enzyme inhibitor (perindopril)	Improve vascular functioning (DMT)	NCT02085265	Recruiting	Sunnybrook Health Sciences Center, ADDF	Mar 2014	Mar 2021
**TEP**	Antiamyloid	Antiemetic; activates transport protein ABCC1	Remove amyloid (DMT)	NCT03417986	Recruiting	Immungenetics AG	Nov 2017	Jul 2021
UB-311	Antiamyloid	Active immunotherapy	Reduce amyloid (DMT)	NCT03531710	Recruiting, extension	United Neuroscience	Aug 2018	Mar 2021
Valacyclovir	Neuroprotective, anti-inflammatory	Antiviral agent	Protects against HSV-1/2 infection and inflammation (DMT)	NCT02997982	Recruiting	Umea University	Dec 2016	Apr 2019
				NCT03282916	Recruiting	New York State Psychiatric Institue, NIH, NIA	Feb 2018	Aug 2022
Xanamem (UE2343)	Neuroprotective	Blocks 11 beta-HSD1 enzyme activity	Decrease cortisol production and neurodegeneration (DMT)	NCT02727699	Active, not recruiting	Actinogen	Mar 2017	Jul 2019

Abbreviations: ABCC1, ATP binding cassette subfamily C member 1; ADCS, Alzheimer's Disease Cooperative Study; ADDF, Alzheimer's Drug Discovery Foundation; AMPA, α-amino-3-hydroxy-5-methyl-4-isoxazolepropionic acid; APOE, apolipoprotein E; APP, amyloid precursor protein; ATRI, Alzheimer's Therapeutic Research Institute; BACE, beta-site amyloid precursor protein cleaving enzyme; cAMP, cycling adenosine monophosphate; CB, cannabinoid; DHA, docosahexaenoic acid; DMT, disease-modifying therapy; FLNA, Filamin A; GM-CSF, granulocyte-macrophage colony-stimulating factor; GSK, glycogen synthase kinase; HSD, hydroxysteroid dehydrogenase; HT, hydroxytriptamine; hUCB-MSCs, human umbilical cord blood derived mesenchymal stem cells; MAPK, mitogen-activated protein kinase; MAPT, microtubule-associated tau; NCCIH, National Center for Complementary and Integrative Health; NIA, National Institute on Aging; NMDA, N-methyl-D-aspartate; PDE, phosphodiesterase; PPAR, peroxisome proliferator-activated receptor; PUFA, polyunsaturated fatty acids; SGLT2, sodium-glucose transporter 2; SV2A, synaptic vesicle protein 2A; TEP, thiethylperazine.

NOTE. Seventy-four agents in 83 phase 2 clinical trials currently ongoing as of February 12, 2019 according to clinicaltrials.gov.

Bolded terms represent new agents into the 2019 phase 2 pipeline.

∗ Reported as terminated or completed after the data collection date of February 12, 2019.

† Phase 1/2 trials.

Of the drugs with amyloid targets, there were seven immunotherapies, one colony-stimulating factor, two BACE inhibitors, and two alpha-secretase modulators. Two agents targeted synaptic activity, two were anti-aggregation agents, and two agents involved neuroprotection or a metabolic MOA. There were two agents targeting both amyloid and tau reduction. Fig. 3 shows the MOAs of agents in phase 2.

Three of the phase 2 trials were prevention trials; 36 trials involved patients with prodromal or prodromal and mild AD; 38 were trials for mild-to-moderate AD; two trials were for patients with severe AD; two included patients with mild, moderate, or severe AD; one included patients with MCI or healthy volunteers; and one trial was for prodromal or mild-to-moderate AD.

Phase 2 trials are shorter in duration and smaller in terms of participant number than phase 3 trials; phase 2 trials had a mean duration of 178 weeks, average treatment period of 45 weeks, and included an average of 143 subjects in each trial.

### 3.4 Phase 1

Phase 1 has 30 agents in 31 trials (Fig. 1, Table 3). There are two cognitive enhancers being assessed in phase 1. There are currently no agents addressing neuropsychiatric symptoms in phase 1. In addition, there are 13 small molecules and 13 biologics being assessed in phase 1. The MOA was not identified for two agents. Two of the small molecules and six of the biologics have amyloid as a primary target or one among several targets. Tau is targeted by one small molecule and four biologics in phase 1 studies. Other mechanisms represented in phase 1 include neuroprotection, metabolic, anti-inflammatory, and regenerative interventions.

**Table 3:** Agents currently in phase 1 of Alzheimer's disease drug development (as of February 12, 2019)

Agent	Agent mechanism class	Mechanism of action	Therapeutic purpose	ClinicalTrials.gov ID	Status	Sponsor	Start date	Estimated end date
**AAVrh.10hAPOE2**	Neuroprotective	Serotype rh. 10 adeno-associated virus gene transfer vector expressing the cDNA coding for human ApoE2	Conversion of the ApoE protein isoforms in the CSF of ApoE4 homozygotes from ApoE4 to ApoE2-ApoE4 (DMT)	NCT03634007	Not yet recruiting	Cornell University	Jan 2019	Dec 2021
Aducanumab∗	Antiamyloid	Monoclonal antibody	Remove amyloid (DMT)	NCT01677572	Active, not recruiting	Biogen	Oct 2012	Oct 2021
**AL002**	Anti-inflammatory	Monoclonal antibody targeting TREM2 receptors	Prevents inflammatory activity (DMT)	NCT03635047	Recruiting	Alector	Nov 2018	Mar 2020
**AL003**	Anti-inflammatory	Monoclonal antibody targeting SIGLEC-3	Reactivates microglia and immune cells in the brain (DMT)	NCT03822208	Not yet recruiting	Alector	Mar 2019	Jul 2020
Allopregnanolone (Allo-IM)	Neuroprotective, metabolic	GABA receptor modulator	Improve neurogenesis (DMT)	NCT03748303	Not yet recruiting	University of Southern California, University of Arizona, Alzheimer's Association	Dec 2018	Dec 2020
BDPP (bioactive dietary polyphenol preparation)	Neuroprotective	Combination of grape seed polyphenolic extract and resveratrol	Prevents amyloid and tau aggregation (DMT)	NCT02502253	Recruiting	Johns Hopkins University, Mount Sinai School of Medicine	Jun 2015	Oct 2019
BIIB076	Anti-tau	Monoclonal antibody	Remove tau and reduce tau propagation (DMT)	NCT03056729	Recruiting	Biogen	Feb 2017	Jul 2019
**CKD-355**	Undisclosed	Undisclosed	Undisclosed	NCT03802162	Not yet recruiting	Chong Kun Dang Pharmaceutical	Feb 2019	Jul 2019
Crenezumab∗	Antiamyloid	Monoclonal antibody targeting oligomers	Remove amyloid (DMT)	NCT02353598	Active, not recruiting	Genentech	Feb 2015	Sep 2023
**CT1812**	Antiamyloid	Sigma-2 receptor antagonist	Reduce amyloid-beta protein-induced synaptic toxicity (DMT)	NCT03522129	Recruiting	Cognition Therapeutics	May 2018	Dec 2019
**Dabigatran**	Neuroprotective	Direct thrombin inhibitor; anticoagulant	Reduce neurovascular damage (DMT)	NCT03752294	Not yet recruiting	University of Rhode Island, ADDF, Boehringer Ingelheim	Nov 2018	Dec 2021
**DNL747**	Neuroprotective, anti-inflammatory	RIPK1 inhibitor	Reduce cytokines and other inflammatory factors (DMT)	NCT03757325	Recruiting	Denali Therapeutics	Feb 2019	Aug 2019
**Efavirenz**	Antiamyloid	Antiretroviral; nonnucleoside reverse transcriptase inhibitor	Increase cholesterol removal and enhance amyloid reduction (DMT)	NCT03706885	Recruiting	Case Western Reserve University, Cleveland Medical Center, Massachusetts General Hospital	Dec 2018	May 2020
Escitalopram & Venlafaxine	Neurotransmitter based	SSRI, SNRI	Improve neurotransmission (cognitive enhancer)	NCT03274817	Recruiting	New York University	Jul 2017	Jan 2019
hMSCs (human mesenchymal stem cells)	Regenerative	Stem cell therapy	Regenerate neurons	NCT02600130	Recruiting	Longeveron	Aug 2016	Mar 2020
Insulin aspart (intranasal)	Metabolic	Increase insulin signaling in the brain	Neuroprotection and enhanced neuronal function; protects against amyloid toxicity (DMT)	NCT02462161	Recruiting	Wake Forest School of Medicine, NIA, General Electric	May 2015	Sep 2019
**J147**	Neuroprotective	Mitochondrial ATP synthase inhibitor	Protects neurons from multiple toxicities associated with aging (DMT)	NCT03838185	Recruiting	Abrexa	Jan 2019	Jan 2020
JNJ-63733657	Anti-tau	Monoclonal antibody	Remove tau and reduce tau propagation (DMT)	NCT03375697	Recruiting	Janssen	Dec 2017	Oct 2019
Lu AF20513	Antiamyloid	Active immunotherapy	Remove amyloid (DMT)	NCT02388152	Active, not recruiting	Lundbeck	Mar 2015	Dec 2019
				NCT03668405	Recruiting, extension	Lundbeck	Jun 2018	Nov 2020
				NCT03819699	Recruiting	Lundbeck	Dec 2018	Jun 2019
LY3002813	Antiamyloid	Monoclonal antibody	Remove amyloid (DMT)	NCT02624778	Active, not recruiting	Eli Lilly	Dec 2015	May 2020
LY3303560	Anti-tau	Monoclonal antibody	Remove tau and reduce tau propagation (DMT)	NCT03019536	Active, not recruiting	Eli Lilly	Jan 2017	Jun 2020
**LY3372993**	Antiamyloid	Monoclonal antibody	Remove amyloid (DMT)	NCT03720548	Recruiting	Eli Lilly	Nov 2018	Sep 2021
**MK-4334**	Undisclosed	Undisclosed	Undisclosed	NCT03740178	Not yet recruiting	Merck	Jan 2019	Jun 2019
NDX-1017	Regenerative	Hepatocyte growth factor	Regenerate neurons (DMT)	NCT03298672	Recruiting	M3 Biotechnology, ADDF, Biotrial Inc.	Oct 2017	Apr 2019
NPT088	Antiamyloid, anti-tau	IgG1-Fc-GAIM fusion protein	Clear amyloid and tau (DMT)	NCT03008161	Active, not recruiting	Proclara Biosciences, Alzheimer's Association	Dec 2016	Apr 2019
Salsalate	Anti-inflammatory	Nonsteroidal anti-inflammatory	Reduce neuronal injury (DMT)	NCT03277573	Recruiting	University of California, San Francisco	Jul 2017	Oct 2019
Telmisartan	Neuroprotective, anti-inflammatory	Angiotensin II receptor blocker, PPAR-gamma agonist	Improve vascular functioning and effects on amyloid pathology (DMT)	NCT02471833	Recruiting	Emory University	Apr 2015	Apr 2019
**THN201**	Neurotransmitter based	Cholinesterase inhibitor + antimalarial glial cell modulator	Improve acetylcholine signaling and modulate astrocyte function (DMT)	NCT03698695	Recruiting	Theranexus	Sep 2018	Jul 2019
TPI-287	Anti-tau	Microtubule protein modulator	Reduce tau-mediated cellular damage (DMT)	NCT01966666	Active, not recruiting	University of California, San Francisco	Nov 2013	Mar 2019
Vorinostat	Neuroprotective	Histone deacetylase inhibitor	Enhance multiple cellular processes including tau aggregation and amyloid deposition (DMT)	NCT03056495	Recruiting	German Center for Neurodegenerative Diseases, University Hospital, Bonn, University of Gottingen	Sep 2017	Oct 2019

Abbreviations: ADDF, Alzheimer's Drug Discovery Foundation; ApoE, apolipoprotein E; BACE, beta-site amyloid precursor protein cleaving enzyme; CSF, cerebrospinal fluid; DMT, disease-modifying therapy; GABA, gamma-aminobutyric acid; GAIM, general amyloid interaction motif; NIA, National Institute on Aging; PPAR, peroxisome proliferator-activated receptor; RIPK1, receptor-interacting serine/threonine-protein kinase 1; SIGLEC-3, sialic acid-binding Ig-like lectin 3; SSRI, selective serotonin reuptake inhibitor; SNRI, serotonin-norepinephrine reuptake inhibitor; TREM2, triggering receptor expressed on myeloid cells 2.

NOTE. Thirty agents in 31 phase 1 clinical trials currently ongoing as of February 12, 2019 according to clinicaltrials.gov.

Bolded terms represent new agents into the 2019 phase 1 pipeline.

∗ Reported as terminated or completed after the data collection date of February 12, 2019.

Phase 1 trials had an average duration of 141 weeks (recruitment and treatment period) and included a mean number of 58 participants in each trial.

### 3.5 Trial sponsors

Across all trials, 54% are sponsored by the biopharma industry, 35% by Academic Medical Centers (with funding from NIH, industry, or other entities), and 10% by others. Table 4 shows the sponsor of agents in each phase of development.

**Table 4:** Trial sponsor for each phase of development (clinicaltrials.gov as of February 12, 2019)

Sponsor	N of trials (%)		
	Phase 1	Phase 2	Phase 3
Biopharma	18 (58)	39 (47)	28 (67)
Academic Medical Centers	8 (26)	20 (24)	7 (17)
NIH	0	0	0
NIH and Academic Medical Centers	0	5 (6)	2 (5)
NIH and Industry	0	2 (2)	1 (2)
Consortium/foundation	0	2 (2)	0
Industry and consortium/foundation	2 (6)	5 (6)	2 (5)
Academic Medical Centers and consortium/foundation	1 (3)	2 (2)	0
Industry, Academic Medical Centers, and consortium/foundation	2 (6)	2 (2)	0
Other combinations	0	6 (7)	2 (5)

Abbreviation: NIH, National Institutes of Health.

### 3.6 Biomarkers

Table 5 shows the biomarkers used as outcome measures in current phase 2 and phase 3 AD clinical trials as described in the federal website; not all trial descriptions in clinicaltrials.gov note if biomarkers are included in the trial.

**Table 5:** Biomarkers as outcome measures in phase 2 and phase 3 trials for agents in the Alzheimer's disease drug development pipeline (clinicaltrials.gov as of February 12, 2019)

Biomarker	N of trials (%)	
	Phase 3	Phase 2
CSF amyloid	14 (33)	15 (18)
CSF tau	13 (31)	17 (20)
FDG-PET	1 (2)	10 (12)
vMRI	10 (24)	8 (10)
Plasma amyloid	5 (12)	5 (6)
Plasma tau	2 (5)	1 (1)
Amyloid PET	11 (26)	6 (7)
Tau PET	8 (19)	2 (2)

Abbreviations: CSF, cerebrospinal fluid; FDG, fluorodeoxyglucose; PET, positron emission tomography; vMRI, volumetric magnetic resonance imaging.

AD biomarkers served as secondary outcome measures in 16 phase 3 DMT trials and 29 phase 2 DMT trials. The most common biomarkers used were cerebrospinal fluid (CSF) amyloid, CSF tau, volumetric magnetic resonance imaging, and amyloid PET. Of the 25 phase 3 DMT trials, five trials (20%) used amyloid PET as an entry criterion, two (8%) used CSF amyloid, and eight (32%) used either amyloid PET or CSF amyloid. Ten (17%) out of 60 phase 2 DMT trials used amyloid PET as an entry criterion, seven (12%) used CSF amyloid, and six (10%) used either amyloid PET or CSF amyloid. Ten DMT trials in phase 3 and 37 in phase 2 did not require biomarker confirmation of AD for trial entry.

Table 5. Biomarkers as outcome measures in phase 2 and phase 3 trials for agents in the Alzheimer's disease drug development pipeline (clinicaltrials.gov as of February 12, 2019)

### 3.7 Devices

A variety of approaches to brain stimulation are under study in clinical trials for AD (Table 6). These range from deep brain stimulation with implanted electrodes to surface application of light, electric current, and laser therapy. Most of the trials target cognitive enhancement; a few trials posit effects on amyloid, tau, inflammation, oxidative stress, or mitochondrial function [20], [21]. Targets have varied from deep brain stimulation of fornix and memory-related structures to surface stimulation of parieto-frontal regions. The few completed studies have shown no consistent cognitive benefit; the techniques have been safe with acceptable adverse event profiles [22]. There are no FDA-defined phases for device trials, and most trials did not list the phase on clinicaltrials.gov. The stages of development for device studies can be divided into pilot, pivotal, and postapproval phases.

**Table 6:** Devices in clinical trials for treatment of Alzheimer's disease (as of February 12, 2019)

Device	Mechanism of action	Clinicaltrials.gov ID	Sponsor
tDCS	Low intensity electric current to modulate cortical excitability and brain plasticity	NCT02772185	Federal University of Paraiba, Brazil
		NCT03638284	Center for Addiction and Mental Health
		NCT03288363	Centre Hospitalier Esquirol
		NCT02873546	Centre Hospitalier Universitaire de Besancon
		NCT02155946	VA Office of Research and Development
Transcranial alternating current stimulation (tACS)	Gamma frequency stimulation to the region of maximum amyloid burden; microglia activation and decrease amyloid and tau depositions	NCT03290326, NCT03412604	Beth Israel Deaconess Medical Center
SonoCloud	Low intensity contact ultrasound implant to open the blood-brain barrier; allows increased intracerebral bioavailability of anti-AD drugs; may also allow endogenous antibodies to penetrate the brain parenchyma and target amyloid plaques even without any adjunct antiamyloid treatment	NCT03119961	CarThera
MemorEM 1000	Transcranial electromagnetic treatment; disaggregation of toxic oligomers; mitochondrial enhancement	NCT02958930	NeuroEm Therapeutics
Neuro Gamma	Photobiomodulation–administers low energy, near-infrared LED light to the brain transcranially and intranasally; reduces oxidative stress and neuroinflammation	NCT03484143, NCT03328195	Vielight
		NCT03160027, NCT03405662	University of California, San Francisco
RGn530	Photobiomodulation device; reduces oxidative stress and neuroinflammation	NCT03672474	University Hospital, Montpellier (device by REGEnLIFE)
Electroconvulsive therapy	Improve cognition by increasing brain-derived neurotrophic factor levels	NCT02438202	Central Institute of Mental Health, Mannheim
ExAblate Model 4000	Blood-brain barrier disruption by focal ultrasound	NCT03671889, NCT03739905	InSightec
GammaSense stimulation system	Visual sensory stimulation device flickering lights at gamma frequency to drive gamma oscillations in brain areas; increase cerebral blood flow and reduce amyloid	NCT03556280	Cognito Therapeutics
		NCT03543878	Emory University, Georgia Institute of Technology
Low level laser therapy	Modulate cellular metabolism and regeneration	NCT02537626	Erchonia Corporation
DBS	Directly target and modulate the activity of brain structures implicated in memory functioning; improve cognition	NCT03347084	University of California, Los Angeles
		NCT03352739	Xuanwu Hospital, China, Beijing Pins Medical Co.
		NCT03622905	Functional Neuromodulation
		NCT03290274	Hospital San Carlos, Madrid
rTMS	Stimulate different areas of the brain to induce changes in brain activity and modify impaired neural networks	NCT03121066	Universitat Oberta de Catalunya
		NCT02908815	University of Manitoba
		NCT03270137	Instituto Nacional de Psipquiatria Dr. Ramon de la Fuente
		NCT02190084	Central Arkansas Veterans Healthcare System
		NCT03778151	Fondazione Santa Lucia
NEUROLITH	TPS consisting of short acoustic pulses with an ultrasound frequency to stimulate the brain; maintains and improves cognitive abilities	NCT03770182	Storz Medical
tVNS	Stimulation of the auricular branch with electrodes on the external ear to improve cognition	NCT03359902	University of Florida, NIA
NeuroAD	Combination of TMS and cognitive training; stimulates areas of the brain responsible for cognitive functions that have been impaired by AD and makes them more receptive to cognitive training	NCT01825330	Neuronix

Abbreviations: AD, Alzheimer's disease; DBS, deep brain stimulation; rTMS, repetitive transcranial magnetic stimulation; tDCS, Transcranial direct current stimulation; tACS, Transcranial alternating current stimulation; TMS, transcranial magnetic stimulation; TPS, Transcranial pulse stimulation; tVNS, Transcutaneous vagal nerve stimulation

NOTE: Thirty-three device trials currently ongoing (“recruiting,” “active, not recruiting,” and “not yet recruiting”) as of February 12, 2019 according to clinicaltrials.gov.

## 4 Discussion

In 2018, the FDA approved 59 novel pharmacotherapies across all therapeutic areas, breaking the 1996 record of 53 drug approvals [23], [24]. There were 42 small molecule therapies and 17 biological therapies approved [24]. Eight new neurological drugs were included among the new therapies: 3 migraine treatments (all were calcitonin gene-related peptide receptor antibodies), 2 for seizures in Dravet syndrome (1 included Dravet syndrome and Lennox-Gastaut syndrome), two for hereditary transthyretin-mediated amyloidosis, and 1 for Fabry disease. The latter three agents can be regarded as DMTs, the others provide relief of symptoms albeit life-threatening symptoms in the case of the epilepsies. The therapies for transthyretin-mediated amyloidosis are RNA-based interventions (antisense oligonucleotide or interference RNA) representing a new approach to neurological disorders. Oligonucleotide-based therapies have shown initial promise in Huntington's disease and may have applications in other neurodegenerative disorders including AD [25]. There is one RNA-based treatment in the AD pipeline (IONIS MAPTRx).

Several agents have completed clinical trials since the time of last year's pipeline analysis and shown no drug-placebo difference. LTMX targeted tau pathology in AD and did not establish efficacy [19]. Azeliragon is a receptor for advanced glycation end products inhibitor and was found to produce no drug-placebo difference in a trial of mild-to-moderate AD. Crenezumab is a monoclonal antibody that targeted oligomeric forms of amyloid-beta protein (Aβ) [26]. It failed to show a drug-placebo difference at the time of a futility analysis in two large clinical trials, and development of the agent was halted. Similarly, aducanumab trials were recently stopped after a futility analysis. Verubecestat is a BACE inhibitor whose development was halted for futility in a mild-to-moderate AD clinical trial [27]. A trial of verubecestat in patients with prodromal AD defined by clinical and amyloid PET measures was halted after a futility analysis suggested that the agent could not succeed. Similarly, lanabecestat did not meet the criteria to continue after a futility analysis. Atabecestat is a BACE inhibitor being assessed in preclinical AD; the trial was discontinued when elevated liver enzymes were observed among some trial participants. Intranasal insulin was assessed in mild-to-moderate AD and showed no drug-placebo difference [28]. Pioglitazone, an insulin sensitizing agent was stopped for futility in a preclinical AD trial. A trial of a FYN inhibitor (AZD0530) used fluorodeoxyglucose PET as the primary outcome and showed no drug-placebo difference on the biomarker or any clinical measure [29]. Fluorodeoxyglucose PET performed well in this multisite trial suggesting it can be used in multicenter trials to show drug-placebo differences with agents that affect brain metabolism. ITI-007 is a multitransmitter agent being developed for the treatment of schizophrenia and was tested in a clinical trial to determine its effect on agitation in AD. No drug-placebo difference was observed in the trial. Of the 17 phase 3 DMTs listed in our 2018 review, eight have been terminated.

GV-971 is a multitargeted molecule that completed a phase 3 clinical trial in China in 2018 [30]. GV-971 has nonclinical evidence of effects on neuroinflammation, amyloid plaques, neurofibrillary tangles, mitochondrial function, and cholinergic function [30]. In a phase 3 trial conducted in China by an international contract research organization, GV-971 showed a statistically significant benefit over placebo on the Alzheimer's Disease Assessment Scale–cognitive subscale [31]; a trend toward improvement was noted on the clinical interview-based impression of change [32]. There was no impact on functional and behavioral measures. The outcomes appear to have met the criteria required for approval by the Chinese FDA, and the agent is under review.

Biomarkers play an increasingly important role in AD drug development. Participant selection, target engagement, disease course prediction, evidence of disease modification, and side effect monitoring all involve biomarkers [33]. The NIA–Alzheimer's Association established the biomarker-based ATN framework for the diagnosis and characterization of AD [7]. This framework will assist in trials with both accurate diagnosis of AD and biological staging of AD relevant to matching the trial population to the MOA of the agent being assessed [8]. Of the disease-modifying trials currently in the AD pipeline, 52 use amyloid imaging and/or CSF to support the diagnosis, 20 have amyloid imaging as an outcome, ten have tau imaging as an outcome of the intervention. In addition to the specific biomarkers included in the ATN framework, evidence is accruing that the plasma amyloid 40/42 ratio corresponds to the presence of cerebral amyloidosis [34] and that plasma neurofilament light is indicative of neurodegeneration [35]. These increasingly available biomarkers will facilitate screening for clinical trials and may have a role in course prediction and assessing treatment outcome.

Design innovations are evident in recent trials of AD therapeutics. Futility analyses were used to terminate development programs for pioglitazone, verubecestat [27], crenezumab, ITI-007, LY3314814 (lanabecestat), and aducanumab. Futility analyses are conducted when the trial is incomplete but when sufficient data are available to predict if continuing the trial could meet prespecified criteria [36].

The Alzheimer's Disease Composite Score (ADCOMS) [37] has been introduced as a cognitive outcome in several development programs including BAN2401, elenbecestat, and xanamem. The ADCOMS is an analytic approach whose score is based on combining scores on items derived from the Alzheimer's Disease Assessment Scale–cognitive subscale, clinical dementia rating, and Mini-Mental State Examination [38] after these tools are administered in the standard way. ADCOMS constituents were derived from trials of patients with MCI that showed the most change over a one-year period to develop a score that is most likely to show a drug-placebo difference in trials of patients with early-stage disease and very limited cognitive deficits.

Bayesian adaptive designs are being implemented in AD trials. These have been used broadly in non-AD trials including development of cancer and diabetes therapies [39], [40]. Adaptive trials are being used in the BAN2401 trial, Dominantly Inherited Alzheimer Network–Treatment Unit [41], European Prevention of Alzheimer Disease initiative [42], and the Intranasal Oxytocin for Fronto-temporal Dementia (FOXY) trial of intranasal oxytocin for frontotemporal dementia [43]. The trial of ABT-089 pioneered the use of an adaptive design in AD [44]. Bayesian designs use data derived from the ongoing trial to inform dose allocation, trial duration, sample size, or response to adverse events; decisions are prespecified before trial initiation. Dose-adaptive designs are participant-centric in that they allow study subjects to be assigned to the doses most likely to succeed or least likely to produce adverse events.

It has been argued that there is no “pipeline” of drug development because agents often do not proceed systematically from phase 1 to phase 3 and irregularities are common [45]. We use the word “pipeline” to categorize agents in early, middle, and late-stage trials. In AD drug development, agents tend to proceed from phase 1 single and multiple ascending dose studies to phase 2 proof-of-concept (POC) studies, and then to phase 3 registration-type trials. Testing for POC in phase 2 depends on dose ranges and safety established in phase 1 and provides the foundation for phase 3. Repurposed agents may have irregular pathways going from approved status for one indication to phase 1 or phase 2 to define dose and POC before testing in phase 3 for the AD indication [46], [47]. The concept of “pipeline” applies as an imprecise but generally accurate overview of drug development for AD.

There are more agents in the AD pipeline in 2019 than was observed in the 2018 pipeline. There are 28 agents in phase 3 (compared with 26 in 2018), 74 agents in phase 2 (compared with 63 in 2018), and 30 in phase 1 (compared with 23 in 2018).

The lack of success in AD drug development has given rise to nihilism with regard to the ability of the field to develop agents that meaningfully modify the progression of AD. Suggestions to abandon the amyloid hypothesis, focus exclusively to combination therapies, place more emphasis on lifestyle interventions to prevent AD or reassess our assumptions and build new models to drive drug development are all voiced, and each of these perspectives have merit. Reviews of the pipeline show that lessons are learned from all trials; even negative and futile outcomes are highly informative and provide guidance for future trials. The overview of trials document a shift toward more diversification of targets between phase 3 and phase 2, the entry of combination therapies into the pipeline, and the use of biomarkers to allow early assessments of the impact of candidate interventions on disease biology.

Several agents have shown no drug-placebo difference, and the development programs have been discontinued. A few programs successfully demonstrated drug-placebo differences in phase 2 and are advancing. Progress depends on innovation and learning from exploration of new targets, assessment of new candidates, and implementation of new trial features. As in other chronic disease such as cancer, human immunodeficiency virus (HIV), and cardiovascular disease, a learning phase preceded periods whose sequential incremental successes led to meaningful treatments.

**Research in context**

1. Systematic review: There is a high rate of failure of drug development for Alzheimer's disease. New treatments are urgently needed, and review of the drug development pipeline can improve our understanding of how best to advance new therapies. We reviewed all drugs currently in clinical trials for Alzheimer's disease listed in the federal government database clinicaltrials.gov.
2. Interpretation: We showed that there are 132 agents in clinical trials for the treatment for Alzheimer's disease. Ninety-six of these drugs are disease-modifying agents intended to change the underlying biology of Alzheimer's disease. Nineteen of the drugs are intended to be cognitive enhancing agents, and 14 are being developed for the treatment of neuropsychiatric and behavioral symptoms. We provide an overview of drugs currently in clinical trials for Alzheimer's disease.
3. Future directions: Progress is being made in terms of defining new targets for the treatment of Alzheimer's disease, developing new agents, introducing innovative clinical trial designs, incorporating a broader range of populations in clinical trials, and developing new biomarkers that provide insight into the impact of emerging therapies. Improvements in drug development success rates are anticipated.
